# Gallic Acid Is the Major Active Component of Cortex Moutan in Inhibiting Immune Maturation of Human Monocyte-Derived Dendritic Cells

**DOI:** 10.3390/molecules200916388

**Published:** 2015-09-10

**Authors:** Ben Chung Lap Chan, Long Fei Li, Shui Qing Hu, Elaine Wat, Eric Chun Wai Wong, Vanilla Xin Zhang, Clara Bik San Lau, Chun Kwok Wong, Kam Lun Ellis Hon, Patrick Chi Leung Hui, Ping Chung Leung

**Affiliations:** 1 State Key Laboratory of Phytochemistry and Plant Resources in West China, Institute of Chinese Medicine, the Chinese University of Hong Kong, Hong Kong, China; E-Mails: benchan99@cuhk.edu.hk (B.C.L.C.); garylfli@cuhk.edu.hk (L.F.L.); cellhu@gmail.com (S.Q.H.); elaine.wat@cuhk.edu.hk (E.W.); cwwong_eric@cuhk.edu.hk (E.C.W.W.); vanilla2334@gmail.com (V.X.Z.); claralau@cuhk.edu.hk (C.B.S.L.); ck-wong@cuhk.edu.hk (C.K.W.); 2 Department of Chemical Pathology, Prince of Wales Hospital, the Chinese University of Hong Kong, Shatin, NT, Hong Kong, China; 3 Department of Paediatrics, Prince of Wales Hospital, the Chinese University of Hong Kong, Shatin, NT, Hong Kong, China; E-Mail: ehon@cuhk.edu.hk; 4 Institute of Textiles and Clothing, the Hong Kong Polytechnic University, Hung Hom, Kowloon, Hong Kong, China; E-Mail: tchuip@inet.polyu.edu.hk

**Keywords:** atopic dermatitis, Cortex Moutan, dendritic cells, gallic acid, high-speed counter-current chromatography, Penta Herb Formula

## Abstract

Atopic dermatitis (AD) is a widely prevalent and chronically relapsing inflammatory skin disease. Penta Herbs Formula (PHF) is efficacious in improving the quality of life and reducing topical corticosteroid used in children with AD and one of the active herbs it contains is Cortex Moutan. Recent studies showed that altered functions of dendritic cells (DC) were observed in atopic individuals, suggesting that DC might play a major role in the generation and maintenance of inflammation by their production of pro-inflammatory cytokines. Hence, the aims of the present study were to identify the major active component(s) of Cortex Moutan, which might inhibit DC functions and to investigate their possible interactions with conventional corticosteroid on inhibiting the development of DC from monocytes. Monocyte-derived dendritic cells (moDC) culture model coupled with the high-speed counter-current chromatography (HSCCC), high pressure liquid chromatography (HPLC) and Liquid Chromatography-Mass Spectrometry (LCMS) analyses were used. Gallic acid was the major active component from Cortex Moutan which could dose dependently inhibit interleukin (IL)-12 p40 and the functional cluster of differentiation (CD) surface markers CD40, CD80, CD83 and CD86 expression from cytokine cocktail-activated moDC. Gallic acid could also lower the concentration of hydrocortisone required to inhibit the activation of DC.

## 1. Introduction

Atopic dermatitis (AD) is a common chronic relapsing disease with high prevalence in children [1]. For a long time, therapeutic strategies of AD have been dominated by the application of local or systemic steroids or other immunosuppressive agents, which have been limited by their potential unwanted local or systemic side effects [2]. Hence, there is considerable interest in the use of traditional Chinese medicines as a potential adjuvant therapy for AD. In our previous clinical studies [3,4,5,6] which recruited children with AD and using a steroid-free concoction termed Penta Herbs Formula (PHF), consisting of a herbal formula containing Cortex Moutan, Cortex Phellodendri, Flos Lonicerae, Herba Menthae and Rhizoma Atractylodis in a weight ratio of 2:2:2:1:2, it was found that PHF is efficacious in improving quality of life and reducing topical corticosteroids use in children with moderate-to-severe AD. PHF was well tolerated by children and the steroid-sparing actions of PHF seem to be multi-targeted. Our laboratory studies showed that PHF could suppress the proliferation of phytohaemagglutinin and Staphylococcal enterotoxin B (SEB)-stimulated peripheral blood mononuclear cells (PBMC) and inflammatory cytokines productions [5]. Inhibitory actions of PHF on histamine and inflammatory cytokines such as TNF-α and CXCL8 from immunologically activated rat mast cells were also observed and it was shown that suppressive effect was mainly contributed by Cortex Moutan [7]. We have also shown that PHF, Cortex Moutan and gallic acid could suppress the *in vitro* activation of AD-related basophils in allergic inflammation [8]. Apart from mast cells, basophils and PBMCs, accumulating evidence indicates that anti-inflammatory drugs target DC on multiple levels including maturation, migration and differentiation [9]. Altered functions of DC in atopic individuals have been observed, especially inflammatory dendritic epidermal cells have been suspected to play a major role in the generation and maintenance of inflammation by their large production of proinflammatory cytokines [10]. Monocytes are one of the precursors of DC and recent studies showed that monocytes rapidly infiltrate to inflamed tissues and mediate DC differentiation and contribute to the pathogenesis of AD [11,12]. To stop the DC maturation from monocytes may offer a novel approach in the treatment of AD. Three active ingredients—gallic acid, paeoniflorin and paeonol—from Cortex Moutan have been identified with immunosuppressive activities on mast cells [13,14,15]. However, no studies of these compounds on DC have been reported. We hypothesize that these active ingredients from Cortex Moutan may interfere the maturation of DC from monocytes with altered cytokine secretions and functional surface markers expression. Besides, corticosteroids have been shown to decrease the number of mature DC and the proinflammatory cytokine concentrations [16]. In our clinical studies, the use of PHF could reduce topical corticosteroids use in patients. Therefore, the aims of the study were to identify and quantify the major active component(s) of Cortex Moutan, which could inhibit DC functions and investigate their possible interactions with conventional corticosteroids on inhibiting the development of DC from monocytes.

## 2. Results and Discussion

### 2.1. Chromatographic Analyses of Cortex Moutan

**Figure 1:**
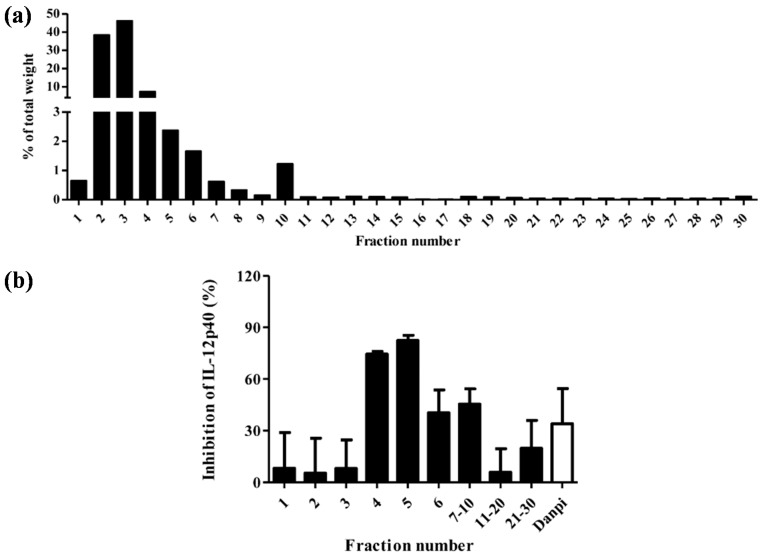
High-Speed Counter-Current Chromatography (HSCCC) of Cortex Moutan. (**a**) The percentage of total weight of 30 HSCCC fractions; (**b**) The effects of the 30 fractions and Cortex Moutan aqueous extract (CM) on IL-12p40 production in monocyte-derived dendritic cells. Values represent mean ± SEM (*n* = 3). Fractions (200 µg/mL) were added to DC and cultured for 48 h.

The dry weights of the 30 fractions of Cortex Moutan are summarized in Figure 1a. Fraction F5 was 640 mg, which represented 7.2% *w*/*w* of the sum of all fractions. As IL-12p40 is the predominant cytokine secreted by activated DC [17], the bioactivity of the Cortex Moutan HSCCC fractions of DC on IL-12p40 were evaluated. 

Figure 1b shows the effects of the 30 fractions (200 μg/mL) and Cortex Moutan aqueous extract (CM) on IL-12p40 production from monocyte-derived dendritic cells (moDC). CM at 200 μg/mL could weakly inhibit IL-12p40 production from moDC. Stronger inhibitory effects of Fractions 4 and 5 (F5) at 200 μg/mL were observed on IL-12p40 production from moDC (71.7% ± 1.8% and 83.3% ± 4.6%, respectively). In contrast, the inhibitory effects of fractions 1 to 3 were very weak, although they constituted up to 85% of the total weight of HSCCC fractions, suggesting most of the inactive compounds had been successfully fractionated by HSCCC. Although potent inhibitory activities on moDC were observed in other fractions, their relative amounts in Cortex Moutan were less than 0.2% *w*/*w* and therefore only fractions 4 and 5 were chosen for further analyses. 

**Figure 2:**
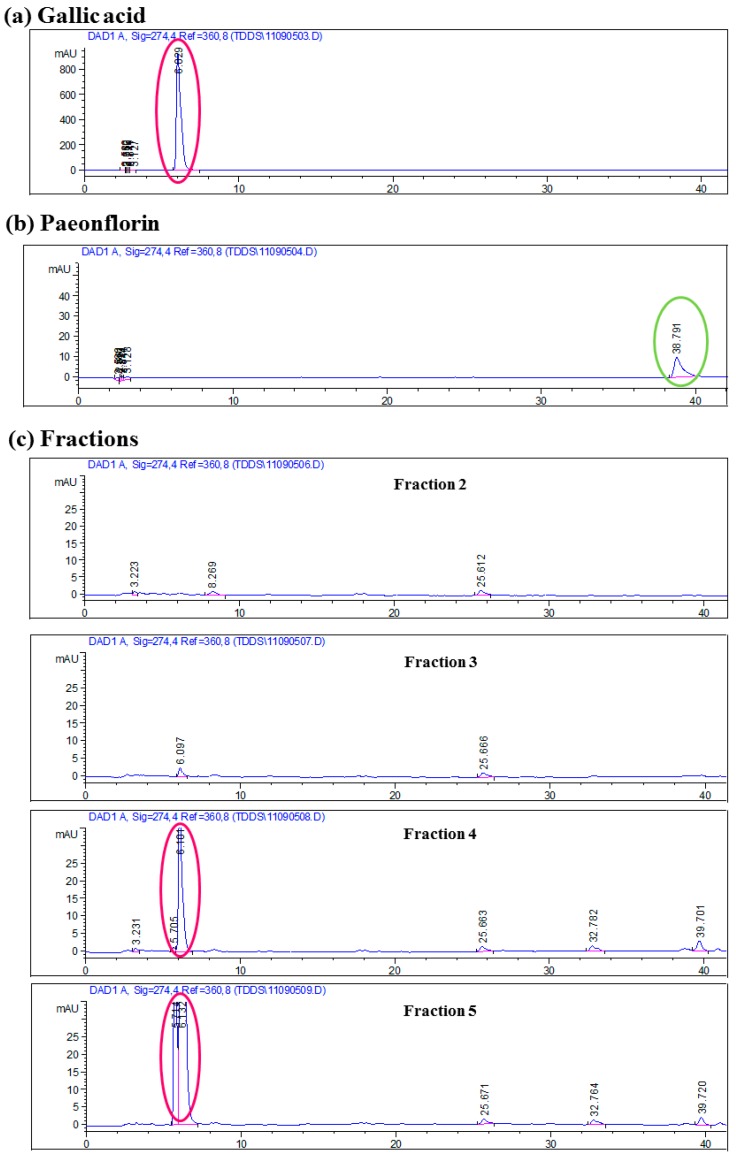
HPLC profiles of bioactive markers (**a**) gallic acid; (**b**) paeoniflorin and (**c**) fractions 2, 3, 4 and 5 of Cortex Moutan HSCCC extract. Detection was performed at UV 274 nm. Representative retention time peaks of the bioactive markers are highlighted in circle. Fractions 4 and 5 showed retention time peak similar to that of gallic acid (highlighted in circle).

For identification and quantification of the presence of active ingredients in F5—gallic acid, paeoniflorin and paeonol—HPLC analyses were performed. Gallic acid and paeoniflorin were shown to be present in fractions 4 and 5 but no paeonol was detected. Figure 2a,b show the HPLC profiles of standards of the bioactive markers gallic acid and paeoniflorin, respectively. The HPLC profiles of the four fractions (fractions 2, 3, 4, and 5) ae shown in Figure 2c. Comparing the HPLC profiles of the fractions with the bioactive markers, both fractions 4 and 5 (fractions with anti-inflammatory activities) showed relatively high peaks at a retention time of 6.0 min, which is the representative retention time peak of gallic acid. However, these retention time peaks are not seen in fractions 2 and 3 (fractions without anti-inflammatory effects). These data therefore lead us to hypothesize that gallic acid is one of the bioactive markers which contributes to the observed beneficial effects of Cortex Moutan aqueous extract. By using LCMS analyses, we further quantified the amount of gallic acid and paeoniflorin in Cortex Moutan fraction F5 as 17.8% and 1.1%, respectively.

### 2.2. Effects of Active Fraction F5 and Its Active Ingredients on Human Monocyte-Derived Dendritic Cells

**Figure 3:**
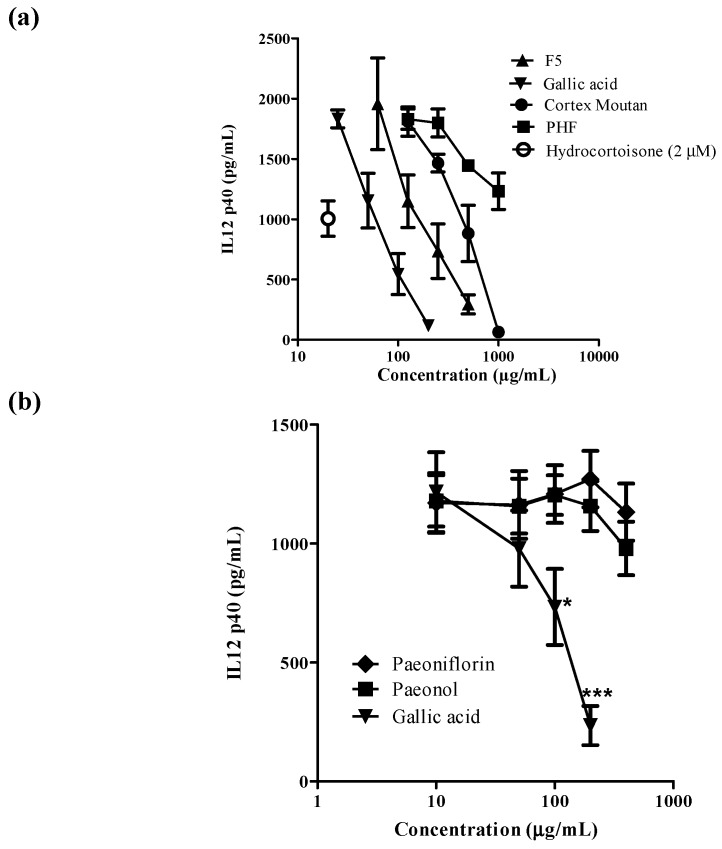
The effects of the active ingredients from Moutan Cortex on IL-12p40 production from human monocyte derived dendritic cells (moDC) (*n* = 3). (**a**) The Effects of fraction 5 (F5) and gallic acid were compared to the crude water extracts of Cortex Moutan and Penta Herb formula (PHF) on IL-12p40 production from human moDC, and hyrodocotroisone (2 μM) was used as positive control; (**b**) The effects of three active ingredients from Cortex Moutan: paeoniflorin, paeonol and gallic acid on IL-12p40 production from human moDC. * and *** indicate *p* < 0.05 and *p* < 0.001, respectively, when compared with the control level of the cytokine production or surface marker expression from cells without incubating with DC-inducing cytokine cocktail.

The pathogenesis of AD is complex and initial studies suggested a T helper cells type-2 (Th2) deviation as the primary reason for the disease [18]. DCs specialize in the uptake, processing, transport and presentation of antigens to immune effector cells in both innate and adaptive immune systems. Given the central role of DCs in immunity and tolerance, they are ideal therapeutic targets for pharmacological modulation of immune responses such as atopic dermatitis [19,20,21]. Under physiological conditions, only very low numbers of DC precursors, like the plasmacytoid and myeloid DC precursors, are present in the peripheral blood, thus hampering research in this area [22]. An alternative approach to produce large number of DCs for investigation is to culture DC from monocytes in the presence of interleukin (IL)-4 and granulocyte-macrophage colony-stimulating factor (GM-CSF) [23]. Furthermore, by modulating the conditions and the duration of DC maturation, monocyte-derived DC with different functions can be obtained and Th2-polarized DC can be induced by adding TNF-α, IL-1β, IL-6 and prostaglandin E_2_ (PGE_2_) for 48 h [24,25,26]. The effects of the water extracts of PHF and Cortex Moutan, active fraction F5 and its active ingredients on IL-12p40 production on moDC were summarized in Figure 3. Hydrocortisone (2 μM) was used as positive control. Dose-dependent inhibitions of IL-12p40 production from activated moDC were observed in Cortex Moutan, F5 and gallic acid (Figure 3a). Gallic acid was the strongest, followed by F5 and Cortex Moutan. The inhibitory effect of PHF was relatively mild. For the three components of Cortex Moutan (Figure 3b), only gallic acid (50–200 μg/mL) was active in inhibiting IL-12p40 from activated moDC, while paeonol and paeoniflorin (10–400 μg/mL) were inactive. Therefore, gallic acid was chosen for subsequent studies.

### 2.3. Effects of Gallic Acid on the Surface Expression of the Functional Markers on Human Monocyte-Derived Dendritic Cells

**Figure 4:**
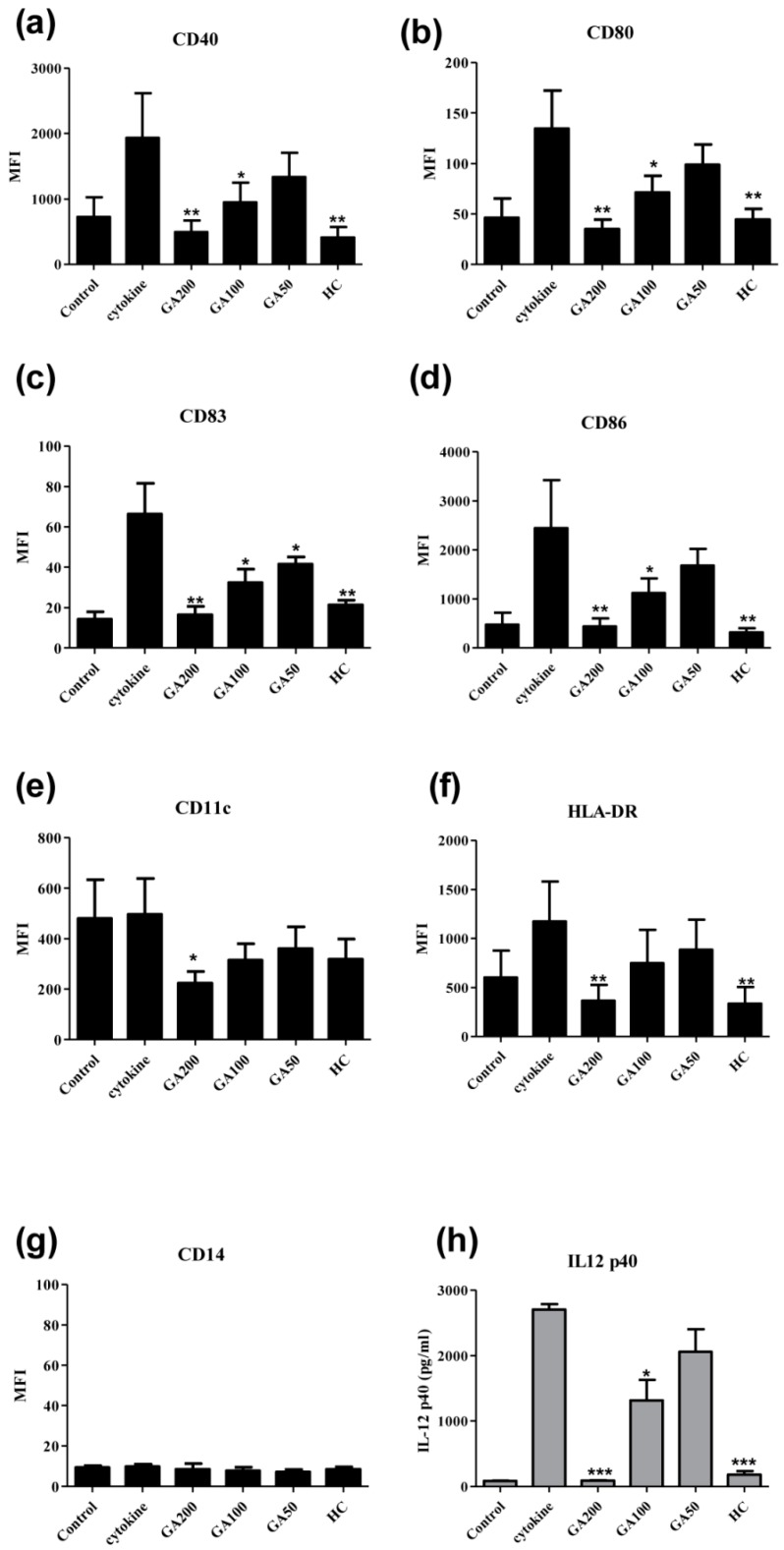
Effects of gallic acid on the surface expression of the functional markers on human monocyte-derived dendritic cells (moDC) (*n* = 4) Control (cells without adding DC-inducing cytokine cocktail); GA200: gallic acid (200 μg/mL); GA100: gallic acid (100 μg/mL); GA50: gallic acid (50 μg/mL); HC: hydrocortisone (2 μM). *, ** and *** indicate *p* < 0.05; *p* < 0.01 and *p* < 0.001, respectively, when compared the level of the cytokine production or surface marker expression from cells without incubated with DC-inducing cytokine cocktail (control). (**a**) CD40; (**b**) CD80; (**c**) CD83; (**d**) CD86; (**e**) CD11c; (**f**) HLA-DR; (**g**) CD14 and (**h**) IL12 p40.

The effects of gallic acid on the expression levels of six surface markers of moDC stimulated with cytokine cocktail [(TNF-α (50 ng/mL), IL-1β (25 ng/mL), IL-6 (1000 U/mL), and PGE_2_ (1 μM)] were studied (Figure 4). Hydrocortisone (2 μM) was used as positive control. CD40 is a co-stimulatory protein found on antigen presenting cells and is required for their activation. CD80 is a protein found on activated B cells and monocytes that provides co-stimulatory signal necessary for T cell activation and survival. CD83 is one of the best-known maturation markers for human DC. It is strongly up-regulated together with co-stimulatory molecules such as CD80 and CD86 during DC maturation. CD86 is a protein expressed on antigen-presenting cells that provides co-stimulatory signals necessary for T cell activation and survival. Gallic acid (100–200 μg/mL) could dose-dependently suppress the expressions of CD40, CD80, CD83 and CD86 (Figure 4a–d), suggesting gallic acid could inhibit the maturation of DCs by those important maturation markers. 

CD11c is a type I transmembrane protein found at high levels on most human DC that induces cellular activation and helps trigger neutrophil respiratory burst. The primary function of HLA-DR is to present peptide antigens, potentially foreign in origin, to the immune system for the purpose of eliciting or suppressing T helper cell responses. Significant suppressive effect of gallic acid on CD11c (Figure 4e) and HLA-DR (Figure 4f) were observed at 200 μg/mL. The expression of CD14 on matured moDC was negative and gallic acid at tested concentration range (50–200 μg/mL) did not modulate the expression of CD14 (Figure 4g). IL-12p40 were also measured (Figure 4h) and gallic acid (100–200 μg/mL) could dose dependently inhibit its production from moDC.

### 2.4. Effects of Gallic Acid with Hydrocortisone on the Development of Human Monocyte-Derived Dendritic Cells

**Figure 5:**
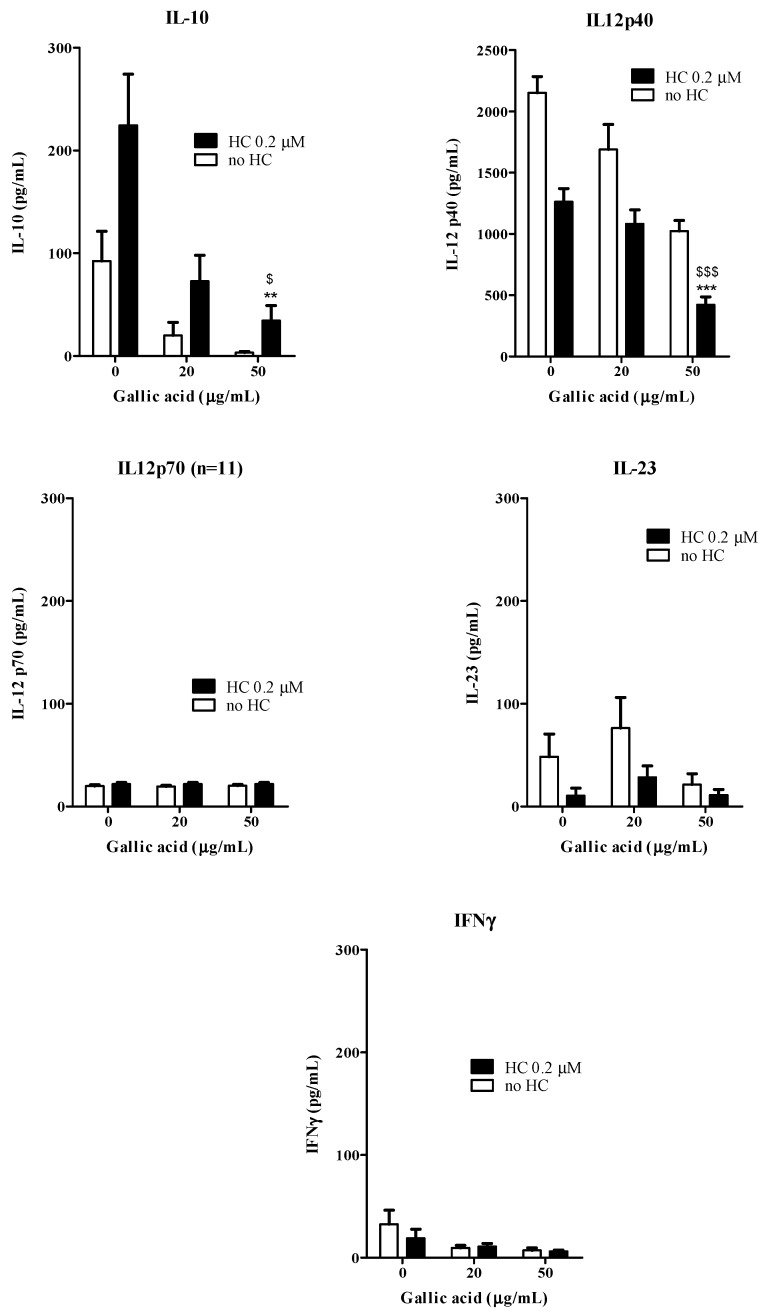
Combined effects of gallic acid and hydrocortisone on the cytokine production from moDC (*n =* 10). The open bars represent the cells were added with gallic acid alone (0, 20 and 50 μg/mL) and the solid bars represent the cells added with gallic acid (0, 20 and 50 μg/mL) and hydrocortisone HC (0.2 μM). ** and *** indicate *p* < 0.01 and *p* < 0.001, respectively, when compared the control level of the cytokine production or surface marker expression from cells without incubating with DC-inducing cytokine cocktail. ^$^ and ^$$$^ indicate *p* < 0.05 and *p* < 0.001, respectively, when compared the control level of the cytokine production or surface marker expression cells with HC and without incubating with DC-inducing cytokine cocktail.

**Figure 6:**
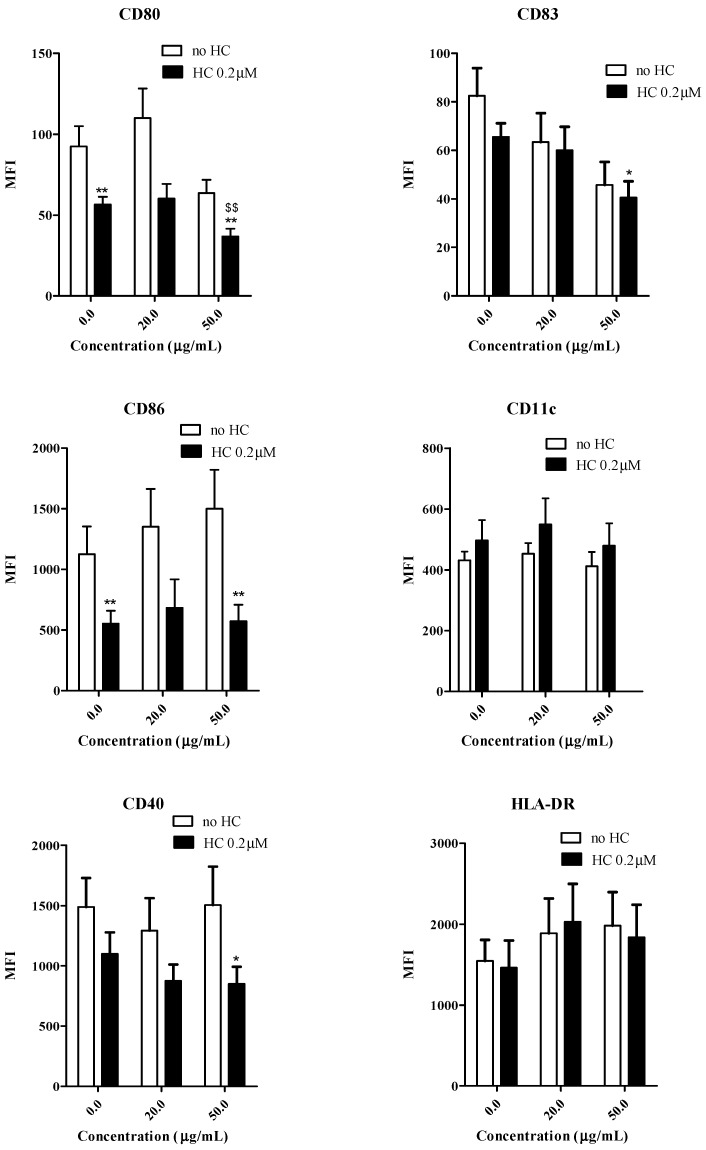
Combined effects of gallic acid and hydrocortisone on the expression of the functional markers from monocyte-derived dendritic cells (moDC) (*n =* 10). The open bars represent the cells were added with gallic acid alone (0, 20 and 50 μg/mL) and the solid bars represent the cells added with gallic acid (0, 20 and 50 μg/mL) and hydrocortisone HC (0.2 μM). * and ** indicate *p* < 0.05 and *p* < 0.01, respectively, when compared the control level of the cytokine production or surface marker expression from cells without incubating with DC-inducing cytokine cocktail. ^$$^ indicate *p* < 0.01, respectively when compared the control level of the cytokine production or surface marker expression cells with HC and without incubating with DC-inducing cytokine cocktail.

Gallic acid (100–200 μg/mL) has been shown to inhibit moDC maturation by down-regulating the DC functional surface markers expression and inflammatory IL-12p40 production. In order to further explore the possible synergistic interactions of gallic acid and corticosteroid in the development of DC from monocytes, sub-optimal dosage of gallic acid (20–50 μg/mL), with hydrocortisone (0.2 μM) were added together to the buffy coat enriched-monocytes for moDC induction process for 6 days. The cells were then washed and maturation of cells were induced by the DC-inducing cytokine cocktail. The levels of IL-12p40, IL-12p70, IL-10, IL-23 and IFN-γ from the culture supernatants were shown in Figure 5. Gallic acid (50 μg/mL) could significantly inhibit the production of IL-10 and IL-12p40 from activated moDC when compared with both drug free control and hydrocortisone alone. Gallic acid and hydrocortisone in combination or used alone did not have any significant modulating effects on IL-12p70, IL-23 and IFNγ production from activated moDC. For surface marker expression (Figure 6), the combined use of gallic acid (50 μg/mL) and hydrocortisone (0.2 μM) could down-regulate the CD40, CD80, CD83 and CD86 expression when compared with the drug free control. Gallic acid has been shown to inhibit pro-inflammatory cytokines from activated mast cells *in vitro* [15]. The current results therefore agree with our hypothesis that the active fraction from Cortex Moutan may interfere the maturation of DCs from monocytes with altered cytokine secretions and functional surface markers expression. The results obtained in this study also agree with our previous clinical findings that PHF is efficacious in improving quality of life by reducing topical corticosteroids use in children with moderate-to-severe AD [4]. Apart from DCs, mast cells are also known for their critical roles in AD attributing to their potent capability to produce multiple pro-inflammatory mediators including histamine, prostaglandins and cytokines after activation [27].

Secondary bacterial infection, especially *Staphylococcus aureus* colonization/infection is also important in the pathophysiology of AD [28]. Gallic acid has been shown to inhibit the growth of *Staphylococcus aureus* and also drug resistant methicillin-resistant *Staphylococcus aureus* (MRSA) [29]. Taken together with the multi-target activities of gallic acid which favor the treatment of AD, it is promising to develop gallic acid with/without corticosteroids in the treatment of AD. As AD involves the interaction among multiple immune cells and for a better study approach, further studies include most sophisticated co-culture models, cell signaling pathways and animal studies are required to warrant the development of the active ingredients from PHF in treatment of AD.

Apart from oral intake, a new treatment for AD using herbal medicine is now in development. Microencapsulation using chitosan-sodium alginate (CSA) is an effective and long lasting method for the release of encapsulated drugs [30]. Recently, bactericidal activities against MRSA were observed in CSA capsulated gallic acid [31]. PHF and Cortex Moutan have been successfully microencapsulated in CSA blend matrix, without cellular toxic effects and could be grafted onto the surface of cotton fabrics [32,33]. For direct contact with the skin of AD patients and sustained controlled release of the herbal extracts, PHF/Cortex Moutan loaded CSA microcapsule may possess potential application in clinical treatment of AD.

## 3. Experimental Section

### 3.1. Authentication and Water Extraction of Cortex Moutan

Cortex Moutan was purchased from Zixun Pharmaceutical Company Limited (Guangzhou, China). The raw herb was extracted by refluxing in boiling water for 2 h and repeating three times to obtain the total water crude extract. The extract was freeze-dried into powder and stored properly in desiccators. Morphological and chemical authentications were accomplished in accordance with the Chinese Pharmacopoeia 2010 [34]. Cortex Moutan was morphologically authenticated by a botanical expert. Besides, chemical authentication was performed using thin layer chromatography (TLC) in accordance to [34]. Briefly, the tested herb (1 g) was powdered and dissolved in diethyl ether (10 mL) and sonicated for 15 min. Mixture was then filtered using cotton wool and evaporated at 60 °C under reduced pressure. The residue was redissolved in methanol (1 mL) and used as the testing herb solution. Powdered reference herb of Cortex Moutan (0.1 g, obtained from National Institute for the Control of Pharmaceutical and Biological Product, Beijing, China) was extracted in the same way as described above and was used as reference herb solution. The chemical reference marker paeonol was prepared by dissolving in methanol. Pre-coated silica gel plates (Silica gel 60 F254, Merck, Darmstadt, Germany) were used for analytical TLC using mobile phase *n*-hexane–ethyl acetate–acetic acid = 40:10:1. TLC profiles were detected by either UV at 254 nm, or by spraying the plate with 2% vanillin/H_2_SO_4_ and heating on a hot plate at 105 °C. The authenticated voucher specimen was deposited in the museum of the Institute of Chinese Medicine, The Chinese University of Hong Kong. The reference markers gallic acid, paeoniflorin and paeonol were purchased from National Institutes for Food and Drug Control, China. Hydrocortisone was purchased from Sigma-Aldrich (St. Louis, MO, USA).

### 3.2. HSCCC Fractionation and Identification of Active Ingredients from Cortex Moutan

Thirty fractions of Cortex Moutan were prepared by repeating the separation condition using a HSCCC TBE-1000 instrument (Tauto, Shanghai, China); a biphasic solvent system (hexane–ethyl acetate–methanol–water = 1:1:1:1); stationary phase:upper phase, mobile phase: lower phase, flow rate: 8 mL/min, sample loading: 80 mL was used. The presence of gallic acid, paeoniflorin and paeonol in F5 was identified and quantified by using high pressure liquid chromatography (HPLC) according to our established conditions as follows: the coil column was first entirely filled with the upper phase of the solvent system at a flow rate of 40 mL/min using a Büchi 615 MPLC pump (Büchi Labortechnik AG, Flawil, Switzerland). Then the apparatus was rotated at 500 rpm, and the lower phase was pumped into the column at the flow rate of 8 mL/min. After the mobile phase front emerged and hydrodynamic equilibrium was established in the column, the sample solution was injected through the injector. When the separation time reached 270 min, the rotation was stopped and all the solution was pushed out of the column by high-pressure gas. The separation temperature was controlled at 25 °C. The effluent from the outlet of the column was continuously monitored at 254 nm and was collected by 10 min/tube and the fractions were freeze-dried into powder for further analyses.

HPLC analyses were performed using a Hewlett Packard Agilent 1100 series HPLC System, equipped with G1329A ALS Autosampler and G1315A Diode Array Detector (Agilent Technologies, San Jose, CA, USA). Sample solution was injected onto an Ultrasphere ODS column (250 mm × 4.6 mm i.d., particle size 5 μm) (Beckman Instrument Inc., Fullerton, CA, USA). All solvents were pre-filtered with 0.45 μm Millipore filter disk (Millipore, Darmstadt, Germany) and degassed. Gradient elution was carried out using the following solvent systems: mobile phase A—acetonitrile; mobile phase B—double distilled water/phosphoric acid (99.0/1.0; *v*/*v*). The flow rate used was 1.0 mL/min and detection was performed at 274 nm. Each sample (10 μL) was injected into the column after filtration through a 0.45 μm filter disk. Identification of the bioactive markers was carried out by comparing the retention times and the UV absorbance of the unknown peaks to those of the standards. Gallic acid (1 mg/mL), and paeonflorin (1 mg/mL) in double distilled water/phosphoric acid 99.7/1 (*v*/*v*) were prepared and analyzed. The system was monitored by a computer equipped with the 32 Karat Software (Beckman Instrument Inc.) for data collection, integration and analysis. To quantify the amount of active ingredients in fraction F5, liquid chromatography–mass spectrometry (LC-MS) analyses were performed using Agilent Technologies 6530 Accurate-Mass Q-TOF LC-MS System. Sample solution was injected onto an Agilent ZORBAX Eclipse Plus C18, 1.8 μm, 3 mm × 100 mm column. All solvents were pre-filtered with 0.45 μm Millipore filter disk (Millipore) and degassed. A gradient elution was carried out using the following solvent systems: mobile phase A—0.1% formic acid in water; mobile phase B—100% acetonitrile. The flow rate used was 0.4 mL/min and detection was performed at negative mode at 169 *m*/*z*. Each sample (5 μL) was injected into the column. Gallic acid (1 mg/mL), and paeonflorin (1 mg/mL) in methanol were prepared and analyzed.

### 3.3. Effects of the CM HSCCC Fractions on the Production of IL-12p40 from moDC in Vitro

The effects of CM HSCCC fractions on lipopolysaccharide (LPS)-activated moDCs were assessed. moDCs (100 μL, 2 × 10^6^ cells/mL) were seeded in 96-well flat bottom microplates and incubated with the HSCCC CM fractions (200 μg/mL) in the presence of LPS (10 ng/mL) for 48 h. The cell free supernatants were collected for IL-12p40 ELISA assays.

### 3.4. Cell Isolation and Generation of Dendritic Cells in Vitro

Mononuclear cells were isolated from buffy coat of healthy adult donors (Red Cross, Hong Kong SAR, China) by Ficoll-Paque Plus density gradient (Amersham Biosciences, Uppsala, Sweden). Monocytes were then isolated from PBMCs by attachment. The cells were plated at 2 × 10^6^ per mL per well in 24-well plate and were allowed to adhere for 45 min, at 37 °C and 5% carbon dioxide (CO_2_). Non-adherent cells were removed by washing the wells two to three times with a gentle stream of medium. Monocytes were then cultured in the presence of two cytokines: granulocyte macrophage colony-stimulating factor (50 ng/mL) and IL-4 (40 ng/mL) at 37 °C under 5% CO_2_. CM and its HSCCC fractions (200 μg/mL), gallic acid, paeoniflorin and paeonol (1–200 μg/mL) with or without corticosteroid hydrocortisone (1–10 μM) were also added to the culture. On day 3, 50% of the medium was replaced with fresh medium and cytokines. DCs were then harvested on day 6 and washed. Maturation of cells were induced by the DC-inducing cytokine cocktail (clinical grade; all media) for 48 h: TNF-α (50 ng/mL), IL-1β (25 ng/mL), IL-6 (1000 U/mL), and PGE_2_ (1 μM).

### 3.5. Flow Cytometric Analysis of Dendritic Cells

On day 8, DCs were harvested, washed and labeled with fluorochrome-conjugated antibodies. After labeling, the cell suspension was washed and re-suspended for flow cytometry. FITC, PE and PE–cyanin 5.1 (PC5)-conjugated isotype controls and CD11c–APC, CD14–FITC, CD40–PerCP, CD80–FITC, CD83–PE, CD86–PE and HLA-DR–APC antibodies (BD Biosciences, San Jose, CA, USA) were used. The DCs were gated to the standard forward-scatter and side-scatter profiles for large cells and the mean fluorescence intensities (MFI) for different cluster of differentiation (CD) markers were normalized with that of RPMI treated negative control as relative fluorescence intensity.

### 3.6. ELISA Assay for Cytokines

The supernatants from DCs cultures were collected after harvesting the cells and stored at −80 °C until assayed for cytokines. The levels of IL-12p40, IL-12p70, IL-10, IL-23 and IFN-γ were then measured in duplicate with human ELISA Kit (BD Biosciences).

### 3.7. Statistical Analysis

Statistical analyses and significance, as measured by the Student’s *t*-test for paired samples or one way analysis of variance (ANOVA) were performed using GraphPad PRISM software version 5.0 (GraphPad Software, San Diego, CA, USA). In all comparisons, *p* < 0.05 were considered as statistically significant.

## 4. Conclusions

Gallic acid is the major active ingredient from Cortex Moutan that can dose dependently inhibit release of IL12-p40 and the functional surface markers expression of CD40, CD80, CD83 and CD86 from cytokine cocktail-activated moDC. Gallic acid could also lower the concentration of hydrocortisone required to inhibit the activation of DC. The current results agree with our hypothesis that gallic acid from Cortex Moutan may interfere with the maturation of dendritic cells from monocytes with altered cytokine secretions and functional surface marker expression. The present study has provided scientific evidence towards the combined use of the major active ingredients gallic acid from Cortex Moutan, together with corticosteroid in the treatment of atopic dermatitis, which may find clinical applications in AD treatment.

## Abbreviations

AD (Atopic dermatitis); CD (cluster of differentiation); CM (Cortex Moutan aqueous extract); DC (dendritic cells); GM-CSF (granulocyte-macrophage colony-stimulating factor); HSCCC (high-speed counter-current chromatography); HLA (human leukocyte antigen); HPLC (high pressure liquid chromatography); IL (interleukin); LCMS (Liquid Chromatography–Mass Spectrometry); MFI (mean fluorescence intensities); moDC (Monocyte-derived dendritic cells); PHF (Penta Herbs Formula); PBMC (peripheral blood mononuclear cells); PGE_2_ (prostaglandin E_2_); T helper cells type-2 (Th2); TLC (thin layer chromatography).
